# Engaging with the Private Sector for Noncommunicable Disease Prevention and Control: Is it Possible to Create “Shared Value?”

**DOI:** 10.5334/aogh.4136

**Published:** 2023-07-03

**Authors:** Téa E. Collins, Svetlana Akselrod, Lina Mahy, Vladimir Poznyak, Daria Berlina, Arian Hatefi, Luke Allen

**Affiliations:** 1 Global NCD Platform, World Health Organization, CH; 2 Multisectoral Action in Food Systems, World Health Organization, CH; 3 Alcohol, Drugs and Addictive Behaviors, World Health Organization, CH; 4 University of California San Francisco, USA

**Keywords:** Noncommunicable diseases, health policy, commercial determinants of health

## Abstract

Noncommunicable diseases (NCDs) are the leading cause of premature mortality worldwide. Corporate interests are sometimes well-aligned with public health, but profiteering from the consumption of products that are known to be the major contributors to the noncommunicable disease burden undermines public health. This paper describes the key industry actors shaping the NCD landscape; highlights the unhealthy commodities’ impact on health and the growing burden of NCDs; and outlines challenges and opportunities to reduce exposure to those risk factors.

Corporations deploy a wide array of strategies to maximize profits at the expense of health, including sophisticated marketing techniques, interference in the policy-making process, opposition and distortion of research and evidence, and whitewashing of health-harming activities through corporate social responsibility initiatives. There can be no shared value for industries that sell goods that harm health irrespective of consumption patterns (such as tobacco and likely alcohol), so government actions such as regulation and legislation are the only viable policy instruments. Where shared value is possible (for example, with the food industry), industry engagement can potentially realign corporate interests with the public health interest for mutual benefit. Deliberate, careful, and nuanced approaches to engagement are required.

## Prioritization of Noncommunicable Diseases in the Global Health Policy Agenda

The 21^st^ century has seen a dramatic transformation of the global disease burden. Noncommunicable diseases (NCDs), including cardiovascular diseases, cancers, chronic respiratory diseases, and diabetes are now the leading causes of mortality globally, with an estimated 41 million deaths per year [1]. More than seventeen million of these deaths are premature, affecting people when they are productive and most likely to have dependents—between the ages of 30 and 69—placing a heavy social and economic toll on societies and threatening sustainable development. Over 86% of premature deaths are taking place in low- and middle-income countries (LMICs), where the health systems are least prepared to provide access to optimal treatment and care [1]. While NCDs are largely preventable, a complex interplay of social, economic, cultural, environmental, and commercial determinants keeps driving the rise of the NCD pandemic.

The rapidly increasing prevalence of NCDs has gained global political prominence following the three United Nations General Assembly High-level Meetings (UNGA HLMs) on the Prevention and Control of NCDs in 2011, 2014, and 2018. At these meetings, there was a broad consensus that the magnitude of the challenge required collaboration with “non-health actors and key stakeholders, where appropriate, including the private sector and civil society, in collaborative partnerships to promote health and to reduce NCD risk factors [2].” The same documents highlight that public policies need to be protected from undue influence from industries that profit from the sale of unhealthy commodities.

Acknowledging the impact of commercial factors on societal health, governments have called on the private sector to strengthen its commitment to national NCD prevention and control efforts by promoting and creating safe and healthy working environments; encouraging reductions in the use of alcohol; taking concrete steps toward eliminating marketing, advertising, and sale of alcoholic products to minors; producing and promoting food products consistent with a healthy diet; reducing children’s exposure to marketing of unhealthy foods and beverages; and improving access to effective medicines and technologies aimed at preventing and controlling NCDs [3].

With the adoption of the 2030 Agenda for Sustainable Development, heads of state and government and other high-level representatives reaffirmed that NCDs are a major barrier to achieving nations’ health and development objectives. The Agenda includes SDG target 3.4 on the reduction of premature mortality from NCDs by one-third and enhanced mental health and well-being by 2030 [4]. Other NCD-related targets encompass strengthening policies and programs to address the use of alcohol and other psychoactive substances and treatment for substance use disorders. These include achieving universal health coverage; scaling up the implementation of the WHO Framework Convention on Tobacco Control; ending all forms of malnutrition, including obesity; supporting research and development; and improving access to vaccines and essential medicines for NCDs. In addition, the 2030 Agenda incorporates SDG 17 on means of implementation, specifically in targets 17.16 on “Global Partnership for Sustainable Development, complemented by multi-stakeholder partnerships that mobilize and share knowledge, expertise, technology, and financial resources” and 17.17 on public, public-private, and civil society partnerships.

The United Nations’ growing emphasis on multistakeholder partnerships and governance, now embedded in the Sustainable Development Goals (SDGs), opened up new opportunities for the private sector to participate in the policy-making processes. Public-private partnerships have become more common in the health sector, as governments and multilateral institutions are applying these arrangements to address diverse health issues [5]. However, the private sector is not homogenous, and a blanket approach to the engagement of the private sector, particularly for NCD prevention and control, may unduly influence the public health agenda [6].

While recognizing that there are many different types of private commercial sector entities playing a significant role in public health, this paper seeks to (i) describe the key industry actors shaping the NCD landscape; (ii) highlight the impact of unhealthy commodities’ production and trade on health and the growing burden of NCDs; and (iii) outline challenges and opportunities associated with engagement with the private sector to reduce the exposure to unhealthy commodities to address NCD prevention and control both globally and nationally.

## Key Industries Shaping the NCD Landscape

The private sector has been defined as ‘organizations that engage in profit-seeking activities and have a majority private ownership, including transnational companies, micro-, small- and medium-sized enterprises, cooperatives, individual entrepreneurs, market vendors, and farmers, who operate in the formal and informal sectors [7]’. The private sector also refers to corporate commercial healthcare providers, which have a large and growing role in health systems worldwide. In some countries, private providers are contracted by governments for basic care towards universal health coverage, such as private general practitioners in the United Kingdom or non-governmental organizations in Bangladesh to provide primary healthcare services. In other systems, private providers deliver additional, beyond-basic healthcare services for those who can afford to pay for them [8].

Apart from the private health service providers, a wide range of industries in both the health and non-health sectors can directly or indirectly influence public health. For example, the contribution of healthcare companies to the research and development of medicines and technologies to prevent, diagnose, treat, and manage health conditions is well established. The industry has both the expertise and business interests in supporting the NCD agenda. As a result, in recent years there has been a significant increase in the number of access to medicines and other global health initiatives involving pharmaceutical companies and their foundations [9]. Other industries, such as sporting goods and fitness, design, and built environments, consumer goods, foods and beverages, health insurance, and media and information technologies are also increasingly becoming pervasive in the health marketplace. For example, personalized health technologies, including wearable devices and smartphone apps, are targeting various chronic diseases and their risk factors, including adherence to medications, remote monitoring, smoking cessation, and mental health [10].

While public-private interactions can address some of the diverse challenges related to NCDs, concerns arise when it comes to agreements between governments and industries that produce products that are negatively associated with health [11]. Commercial determinants of health, here defined as the private sector activities that affect people’s health positively or negatively, come into complex interplay with the social determinants of health, including *intermediate* determinants (e.g., education, occupation, income, ethnicity, race, access to healthcare) and *structural* determinants (e.g., socio-economic and political context), and affect health inequities and individuals’ health throughout the life course [12].

In this context, a major challenge for NCD prevention and control is the influence of transnational corporations that directly or indirectly profit from the consumption of their products (tobacco, alcohol, and unhealthy foods and beverages) proven to influence the NCD risk factors and worsen the populations’ quality of life and health outcomes. Ultimately, the rise of NCDs is a manifestation of a global economic system that currently prioritizes corporate wealth creation over health creation. Many problems and solutions to address the risk factors for health lie outside the health sector, in the domains of finance, trade, agriculture, and investment policies [13].

Understanding that immense threats to health in the modern era cannot be tackled by governments alone, and also recognizing the challenges of reconciling public and private interests, in 2011, Michael Porter and Mark Kramer introduced a new approach for businesses to highlight the importance of creating shared value by identifying and addressing societal challenges, including in the areas where health outcomes intersect with business. The shared value framework seeks to “create new opportunities for companies, civil society organizations, and governments to leverage the power of market-based competition in addressing social problems [14].”

From the NCD perspective, “Big Tobacco,” “Big Alcohol” and “Big Food” are the major industries associated with the production of unhealthy commodities affecting NCDs. The transnational corporations in these sectors operate at all levels (local, national, regional, and global) and have tremendous economic power at their disposal to influence policy processes [15]. Many of them are economies larger than those of national states [16]. For example, the combined revenues of ten large food corporations (Nestlé, PepsiCo, Unilever, Mondelez/Kraft, Coca-Cola, Mars, Danone, Associated British Foods, General Mills, and Kellogg’s) reached more than a billion dollars a day in 2017–2018: in the same period, annual sales of Nestlé, with its over 2000 brands, amounted to USD 91.2 billion, which is roughly the same as the gross national products of Sri Lanka or Kenya [17]. The breastmilk substitute marketing industry is worth $55 billion [18] and the value of the global tobacco and alcohol markets is even more staggering; in 2017, the tobacco industry was estimated to be worth around US$785 billion (*excluding* the world’s largest tobacco company, China Tobacco) while the global 2021 alcoholic drinks market size was estimated to be valued at around $1.5 trillion and projected to nearly double by 2028 [19,20].

Not surprisingly, large corporations in these spheres are increasingly shaping the complex global NCD landscape while the demand for their products continues to grow in LMICs. Given the significant contribution of these products and associated practices to NCDs, is there room for public-private interaction to create shared value and promote population health?

## Unhealthy Commodities’ Impact on Health and the Growing Burden of NCDs

Tobacco continues to be one of the leading causes of premature mortality globally, killing over 8 million people each year. More than 7 million of those deaths are the result of direct tobacco use, while around 1.2 million are due to non-smokers being exposed to second-hand smoke. All forms of tobacco are harmful, and there is no safe level of exposure to this product. Almost half of all children globally regularly breathe air polluted by tobacco smoke in public places, and 65,000 die each year from illnesses attributable to second-hand smoke [21].

Alcohol-related harm has a significant impact on global health. The World Health Organization estimates that alcohol consumption contributed to three million deaths in 2016, of which 1.7 million were related to NCDs, including mental health disorders [22]. Consumption of even one alcoholic drink per day increased the risk of various cancers, including female breast, oral cavity, pharynx, and esophagus [23]. The health, social, and economic risks of alcohol consumption go beyond NCDs. Alcohol use is also linked to violence, injuries, infectious diseases, and harm to others [24]. Recent evidence has questioned the claim that there is an “optimal” level of alcohol consumption for cardiovascular health, suggesting that previously reported risk reductions from light alcohol consumption may be better explained by genetics and other behaviors [25]. The preponderance of evidence suggests that the optimal level of alcohol consumption for most demographic groups is zero alcohol consumption [26].

The synergistic effects of tobacco smoking and alcohol consumption on cancer mortality have also been well documented. Combined consumption of tobacco and alcohol has been linked to a greater effect on cancer than consuming either of these products alone. Heavy drinking also appears to be a risk factor for heavy smoking and vice versa [27].

Unlike tobacco and alcohol, the influence of the food and beverage industry on health is not necessarily negative. Healthy nutritious food is essential for life. Many foods need to be processed to some extent to make them safe and widely available to consumers e.g., drying, packaging, chilling and freezing, and pasteurizing. The challenge is the rapid penetration of global markets by highly-processed foods and drinks that are made from industrially processed substances derived from high-yield crops, such as hydrogenated oils and fats, refined sugars, syrups, and starches, protein isolates, and remnants of intensively reared animals. These foods are often energy-dense products, low in dietary fiber, micronutrients, and phytochemicals [28]. However, they often look, smell, and taste good due to a combination of flavors, colors, thickeners, sweeteners, emulsifiers, sodium, and other additives that make them appealing to consumers, particularly children [29].

**Figure 1:**
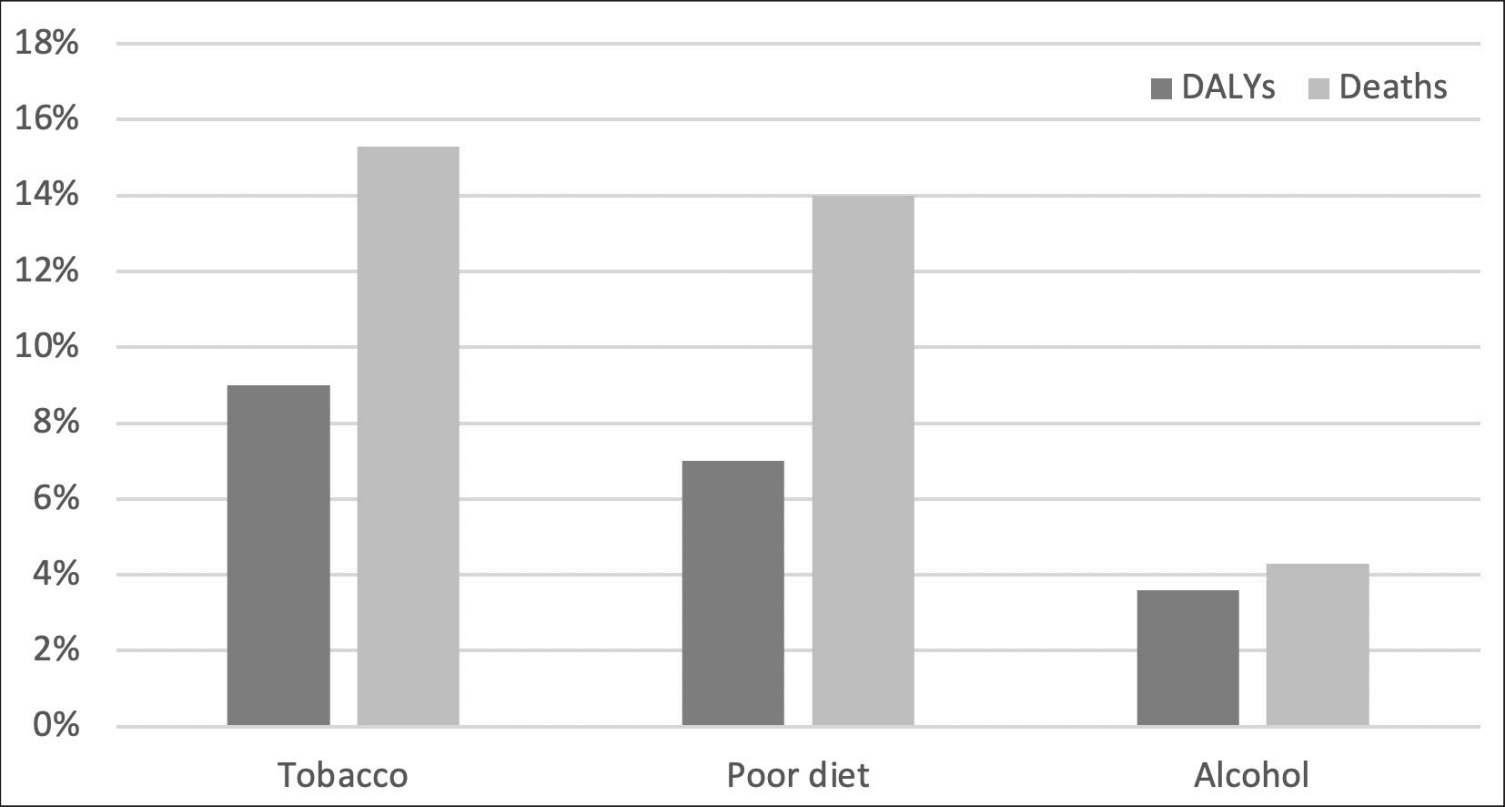
Proportion of All Global Deaths and DALYs Attributable to Tobacco, Poor Diet, and Alcohol.

Approximately 8 million global deaths annually are attributable to unhealthy diets (Figure 1) [30]. Consumption of highly-processed foods and carbonated and other sugary drinks have been linked to the deterioration of the overall nutritional quality of diets and associated with obesity, gastrointestinal disorders, metabolic syndrome, hypertension, diabetes, cardiovascular diseases, various cancers, depression, and other chronic conditions. This is well documented in studies from Brazil, Chile, Columbia, Mexico, the USA, Canada, the UK, France, Belgium, Australia, New Zealand, and Japan [15]. Added sugars in particular, such as fructose-containing sweeteners, sucrose (table sugar), high-fructose corn syrup, maple syrup, and agave nectar, are the most addictive components driving the NCD risk. It is estimated that sugar-sweetened beverages are associated with 184,000 deaths every year globally [31]. In addition, the synergy of high fat with high sugar is extremely effective at stimulating the overconsumption of certain types of foods, especially fast foods. In the USA, 56% of the diet consists of highly-processed foods, and some form of sugar has been added to 74% of the items in American supermarkets [32].

The problem is not confined to Western countries alone. Once high-income country market saturation happens, the food industry tends to shift to lower-income countries. For example, from 2002 to 2016, sales of highly-processed foods increased from 20% in the Latin America and Caribbean region to around 90% in South and Southeast Asia [33]. Young children in Nepal are consuming unhealthy snack food and beverage products in alarming amounts: On average these foods contribute to nearly 25% of the dietary energy intake for children 12 to 23 months, with the highest consumers receiving nearly 50% of their calories from these products [34].

## Challenges

Reducing the harms brought about by tobacco use, unhealthy diets, and alcohol consumption worldwide continues to be a major challenge, particularly in LMICs. It is well documented that transnational corporations use similar tactics and strategies to enhance their sales and undermine public health interventions [20]. Some of these strategies include:

**1. Sophisticated marketing techniques**—Like Big Tobacco, the Big Alcohol industry has substantially expanded its market in LMICs in the past decades, owing in large part to their successful marketing strategies targeting population groups that typically have lower levels of alcohol consumption, including women and young people. For example, a study of 11- to 16-year-old students in Zambia found that students who reported ever receiving a free alcoholic drink from an industry representative had more than 40% higher odds of experiencing alcohol-related behavioral problems (e.g., skipping school or engaging in fighting) and drinking to intoxication versus students who have not received a free drink [35]. Another example refers to an alcoholic beverage that was developed exclusively for women as a ‘stylish’ alternative to beer. The company in question also designed mini liquor bottles to make sure the small size and lower price were attractive to young people and were also easier to transport to rural areas [35]. There is documented evidence that 22% of five-year-old children in Brazil, India, Nigeria, Pakistan, China, and Russia were able to correctly identify a major Philip Morris International brand [36]. A study in Uganda showed that there is widespread advertising of unhealthy foods and beverages around primary and secondary schools (with 86% featuring unhealthy products) [37].

**2. Interference in public policy-making processes**—The tobacco industry’s interference in public health policies is an issue in rich and poor countries alike. For example, Switzerland, which is not a party to the WHO FCTC, allows tobacco company employees to be elected as members of the Federal Assembly while keeping their jobs. Similarly, the governments of Bangladesh, India, and Malaysia continue to hold their shares in tobacco companies. These practices contrast with the decision of the Norwegian government’s pension fund, which stopped investing in tobacco after introducing the fund’s ethical guidelines. Overall, five countries that remain non-parties of the WHO FCTC—Argentina, the Dominican Republic, Indonesia, Switzerland, and the USA—faced more interference from the tobacco industry in undermining national tobacco control efforts than party states [38]. Highly-processed food industry actors also attempt to influence public health policy through coalition management, involvement in policy formulation, legislative preemption, cooption of the policy process, and information management [39,40]. Work by Allen et al. has found that countries with the greatest legislative opportunities for corporations to use their financial resources to directly influence policymaking have the lowest levels of NCD policy implementation, and *Corporate Financial Influence* explains a fifth of the overall variance in global NCD policy implementation [41].

**3. Opposing evidence-based practices and biasing research evidence**—Unhealthy commodities industries often use evidence to undermine public health and oppose regulation. For example, published articles sponsored by the food and beverage industry are four to eight times more likely to have conclusions favorable to the financial interests of the sponsoring companies [42]. The ultra-processed food and beverage industry claims that regulation to address diet-related NCDs does not have the desired effect. It also questions the links between sugar consumption and obesity and a range of NCDs, despite the mounting evidence that the opposite is true [43].

**4. Creating the illusion of corporate social responsibility or shared value**—The WHO FCTC is clear that the tobacco industry cannot be seen as a partner in health-related matters. Recognizing the “fundamental and irreconcilable conflict between the tobacco industry’s interests and public health policy interests,” the Treaty calls on Parties, among others, to “reject partnerships and non-binding or non-enforceable commitments with the tobacco industry [44].”

To shift attention away from the protection of public health and improve its public image, the tobacco industry uses ‘corporate social responsibility’ (CSR) strategies such as supporting tobacco farmers or donating funds to COVID-related activities, poverty alleviation, disaster relief, and sponsoring sports events [28]. Phillip Morris currently holds a one-third stake in a company that is producing a COVID vaccine [45]. Actors within the food industry are also increasingly framing themselves as “part of the solution,” whilst continuing to pursue corporate strategies that prioritize profits over health [46].

In South Africa, the alcohol industry established an Association for Responsible Alcohol Use and is actively sponsoring events such as educational workshops and initiatives to stop underage drinking. Similarly, major food manufacturers in South Africa have active CSR programs focusing on nutrition education [47].

The situation is further compounded by the fact that transnational corporations operate at all levels from global to local and benefit from the opportunities that globalization, trade liberalization, and digitization bring [48]. Irrespective of this fact, governments have a range of valuable tools at their disposal to counter the unhealthy commodity industries’ efforts to advance their business interests at the expense of public health.

## Opportunities to Reduce Exposure to Unhealthy Commodities

### 1. Industry engagement

Public engagement with corporations has a fraught track record but remains an underutilized tool for achieving NCD response goals. Cooperative strategies can leverage diverse interests and assets among stakeholders to promote societal benefit. In the case of the commercial determinants of health, engagement aims to reinforce confluences of public health and corporate interests (e.g., food fortification, health care access initiatives), and to realign interests when in conflict (e.g., to reformulate food or to shift from fossil fuels to clean energy sources).

Foundational to effective engagement is confidence, which arises from a clear understanding of actor interests and consequent behaviors. Public health and corporate actors’ behavior is shaped by different paradigms, the former being responsive to moral and ethical constructs and the latter predominately to profit maximization. Engagement can be undermined when these vastly different paradigms are conflated under the guise of a shared objective when no such shared objective exists (e.g., when more health does not also mean more profit) [49]. Instead, effective engagement requires *ex-ante* identification of how values do or do not align, includes all relevant stakeholders, and establishes with due diligence clear terms of engagement to disclose and manage conflicting interests [50]. Due diligence promotes confidence, safeguarding the health impacts of consumption and engendering trust in the integrity of the policy-making and governance process.

Partnership as a form of engagement is not a viable pathway without shared value, explaining the limited confidence in and effectiveness of public-private partnerships for public health objectives with industries like tobacco, alcohol, and firearms. The public health response to these corporate interests requires a suite of alternative options, including regulation, legislation, and taxation [51].

Given that more alcohol consumption is not associated with more health, and engagement is only appropriate around measures to reduce the harm that require industry participation, given their core roles as developers, producers, distributors, marketers, and sellers of alcoholic beverages [52]. In most other cases, such as with health care goods, food, energy, and social media, among others, shared value creates the possibility for developing win-win strategies that include both better health (or less harm) *and* more profit.

Finally, whilst the most important cooperation takes place within the national sphere, we identify three key supranational actors that can do more at the global scale to rationalize multinational corporate influence on health. First, the World Trade Organization has low priority given to health dimensions of trade and limited expertise in health despite its significant power in promoting global health through its role in regulating and facilitating trade agreements and policy [53]; this is a remediable gap, either through organizational policy or coalition-building. Second, the International Finance Corporation largely deals with private sector investment for development, yet its health scope is mainly limited to private healthcare providers; a broad framework for applying health co-benefits to private sector investment decisions could dramatically shape LMIC norms relating to the commercial determinants of health. Last, the World Economic Forum has significant corporate influence and convening power, yet its focus on digital transformation in healthcare might best be complemented by greater topical attention to corporate influence on health among non-healthcare corporate actors.

### 2. Independent government action

Whilst voluntary action and cooperation are welcome, the health gains that they deliver are only valuable if they do not “undermine, forestall or dilute” more effective actions, such as those that would be achieved via mandatory regulation or independent government action [54]. Building on the WHO Global Coordination Mechanism for NCDs working group recommendations [55], Allen has recommended working with alcohol and food actors to reduce consumption of alcohol and highly-processed foods only for carefully circumscribed issues, through controlled and independently monitored mechanisms, to meet prespecified targets over which industry has no input [56]. As such, a large part of tackling commercial determinants of NCDs depends on unilateral state action.

Effective national laws and regulations are required to implement successful national NCD prevention policies and plans. In this context, the WHO-endorsed “best buys” and other recommended interventions for NCD prevention and control are affordable and feasible in all country settings, provided that there is political leadership, strong ministries of health, and an adequately trained health workforce, as well as secure funding. To be more specific, the implementation of the best-buy interventions to address the main NCD risk factors—tobacco, alcohol, unhealthy diets, and physical inactivity—and scale up treatment to reach 50% coverage by 2030, will cost about US$0.62 per capita in low-income countries and US$1.44 per capita in middle-income countries, which is an average of US$1.27 per year for every person in every country [57].

Implementing WHO-recommended interventions for the major NCD risk factors will also require effective use of law and strong legal and regulatory capacities, as only government-led public regulation can protect public health and improve the NCD burden (Table 1).

**Table 1:** WHO-backed policy options and cost-effective interventions for NCD prevention and control [58].


**Tobacco**	Consider implementing the measures set out in the WHO FCTC and its guidelines for implementation, as well as the Protocol to Eliminate Illicit Trade in Tobacco Products, if applicable, as the foundational instruments in global tobacco control Increase excise taxes and prices on tobacco products Implement large graphic health warnings on all tobacco packages, accompanied by plain/standardized packaging Enact and enforce comprehensive bans on tobacco advertising, promotion, and sponsorship Eliminate exposure to second-hand tobacco smoke in all indoor workplaces, public places, and public transport Implement effective mass media campaigns that educate the public about the harms of smoking/tobacco use and second-hand smoke, and encourage behavioral change Provide cost-covered effective population-wide support (including brief advice, national toll-free quitline services, and mCessation) for tobacco cessation to all tobacco users Provide cost-covered effective pharmacological interventions to all tobacco users who want to quit, through the use of nicotine replacement therapy, bupropion, and varenicline. Establish a tracking and tracing system to support the elimination of illicit trade in tobacco products that is in line with Article 8 of the Protocol to Eliminate Illicit Trade in Tobacco Products Ban cross-border tobacco advertising, promotion and sponsorship, including those through modern means of communication

**Alcohol**	Implement applicable recommendations in WHO’s Global strategy to reduce the harmful use of alcohol through multisectoral actions in the recommended target areas Implement WHO’s global action plan on alcohol 2022–2030 to support and complement policy measures and interventions implemented at the national level following 10 areas recommended in the global strategy to reduce the harmful use of alcohol Strengthen leadership and increase commitment and capacity to address the harmful use of alcohol Increase awareness and strengthen the knowledge base on the magnitude and nature of problems caused by the harmful use of alcohol through awareness programs, operational research, improved monitoring, and surveillance systems Increase excise taxes on alcoholic beverages Enact and enforce bans or comprehensive restrictions on exposure to alcohol advertising (across multiple types of media) Enact and enforce restrictions on the physical availability of retailed alcohol (via reduced hours of sale) Enact and enforce drink-driving laws and blood alcohol concentration limits through sobriety checkpoints Provide brief psychosocial intervention for persons with hazardous and harmful alcohol use Carry out regular reviews of prices concerning the level of inflation and income Establish minimum prices for alcohol where applicable Enact and enforce an appropriate minimum age for the purchase or consumption of alcoholic beverages and reduce the density of retail outlets Restrict or ban promotions of alcoholic beverages in connection with sponsorships and activities targeting young people Provide prevention, treatment and care for alcohol use disorders and comorbid conditions in health and social services Provide consumers with information, including labels and health warnings, about the contents of alcoholic beverages and the harms associated with alcohol consumption

**Diets**	Implement WHO’s Global Strategy on Diet, Physical Activity and Health, the Global strategy for infant and young child feeding jointly developed by WHO and UNICEF and WHO’s Comprehensive implementation plan on maternal, infant, and young child nutrition Develop and implement national nutrient- and food-based dietary guidelines, as well as nutrient profile models for different applications as appropriate Introduce reformulation policies for healthier food and beverage products (for example, elimination of trans-fatty acids and/or reduction of saturated fats, free sugars and/or sodium) Front-of-pack labeling as part of comprehensive nutrition labeling policies for facilitating consumers’ understanding and choice of food for healthy diets Public food procurement and service policies for healthy diets (for example, to reduce the intake of free sugars, sodium and unhealthy fats, and to increase the consumption of legumes, whole grains, fruits and vegetables) Behavioral change communication and mass media campaigns for healthy diets (for example, to reduce the intake of energy, free sugars, sodium, and unhealthy fats, and to increase the consumption of legumes, whole grains, fruits, and vegetables) Policies to protect children from the harmful impact of food marketing on diet Protection, promotion, and support of optimal breastfeeding practices Taxation on sugar-sweetened beverages as part of fiscal policies for healthy diets Subsidies on healthy foods and beverages (for example, fruits, and vegetables) as part of comprehensive fiscal policies for healthy diets Menu labeling in food service for healthy diets (for example, to reduce the intake of energy, free sugars, sodium, and/or unhealthy fats) Limiting portion and package size for healthy diets (for example, to reduce the intake of energy, free sugars, sodium, and/or unhealthy fats) Nutrition education and counseling for healthy diets in different settings (for example, in preschools, schools, workplaces, and hospitals)


Reducing the exposure to risk factors will require whole-of-government commitment and the involvement of government ministries beyond health for policy coherence, and accountability. A stronger leadership role for ministries of health and greater participation of civil society deserves special attention. Professional associations, parliamentarians, academic institutions, patients’ advocacy groups, human rights, and legal organizations all have a role to play to provide advocacy for government actions based on the best available evidence.

Safeguarding populations’ health usually takes place at the national level, but as transnational corporations are part of the global economic system, the need for international collective action to address the NCD pandemic has been growing. At the global level, there have been calls from researchers, professional organizations, and some governments for a binding international treaty to reduce the harmful use of alcohol and related problems, similar to the WHO FCTC [29]. This will require strong political commitments from governments to address industry interference, strengthen the leadership role of WHO in global health, and recognize the damaging impact of the commercial determinants of health [16].

## Conclusions

NCDs are the leading cause of global death and disability, with the tobacco, alcohol, and food and beverage industries playing significant roles in shaping this unfolding slow-motion pandemic. Despite the growth of public-private partnerships in the health sector to promote development objectives, governments need to remain vigilant about not compromising public health interests when engaging with the private commercial sector. For the most part, shared value can only be attained when both parties have something to gain, i.e., health improvements and increased shareholder value through financial returns, although sometimes shared value can be less financially explicit for corporations (e.g., through reduced reputational risks). For that reason, it is absolutely clear that there is no possibility of creating shared value with the tobacco industry. Whilst the alcohol industry appears keen to engage around themes of “responsible drinking,” it is also becoming clear that alcohol consumption is always associated with health risks. As such, inherent conflicts of interest should preclude partnerships and collaboration with governments and health actors in the development of alcohol policies and high-impact strategies and interventions to reduce the harmful use of alcohol.

Engagement with the food and beverage industry needs more careful examination to assess the health impact of their actions and engagements. As highly-processed food producers are quick to point out “there is no such thing as unhealthy foods, only unhealthy diets [59].” The food industry defies binary categorization (friend nor foe), and we would argue that industry actors can be involved in specific public health actions given that essential safeguards are put in place [60].

Beyond the food, tobacco, and alcohol sectors, there are multitudes of commercial actors whose business models do not inherently conflict with public health goals. If carefully handled, there may be huge scope for increasing engagement to tackle NCDs and address broader sustainable development issues—and the SDGs recognize this fact [61]. With the right governance mechanisms, regulatory frameworks, and geographically, politically, and culturally specific policies responsive to disease burden, creating a shared value to address NCDs and their risk factors is a goal for all relevant actors to strive for.

## Data Accessibility Statement

All data used in this article are publicly available.
